# Treatment Compliance in the Long-Term Paranoid Schizophrenia Telemedicine Study

**DOI:** 10.1007/s41347-017-0016-4

**Published:** 2017-05-18

**Authors:** Marek Krzystanek, Krzysztof Krysta, Katarzyna Skałacka

**Affiliations:** 1 Department of Psychiatric Rehabilitation, Medical University of Silesia, ul. Ziolowa 45/47, Katowice, Poland; 2 Department of Psychiatry and Psychotherapy, Medical University of Silesia, ul. Ziolowa 45/47, Katowice, Poland; 3 Institute of Psychology, University of Opole, Opole, Poland

**Keywords:** Schizophrenia, Telepsychiatry, Compliance

## Abstract

Schizophrenia is a chronic disease with a relatively high relapse rate. Different methods are introduced to improve compliance of the patients treated by psychiatrists; among them, a new and promising attitude is telepsychiatry. The 12-month clinical study of the compliance in paranoid schizophrenia was performed on the group of 199 patients. Every patient in the study was given a smartphone with the preinstalled original telemedicine platform (Telemedicine MoneoPlatform). The telemedicine system recorded every confirmation of the drug intake, and according to that, the compliance was counted as the percent of the doses of medication confirmed in relation to the planned ones. In the first month of the study, patients confirmed only 47.6% of the doses as taken. When analyzed in the total group, the compliance significantly decreased over the 12-month period (*p* < 0.001). The compliance observed in our study is lower in comparison with short-term studies, but to our knowledge, this project is the biggest long-term study of the treatment compliance in schizophrenia, performed on a large number of patients, and a possible conclusion is that the adherence in longer lasting project depends highly on the engagement of both patients and psychiatrists.

The course of schizophrenia, number relapses, social functioning, and final outcome depends largely on the compliance to the treatment (Bitter et al. 2015). In this progressive and long-term disease, around 60% of patients stop the treatment after 2–3 months without the consultation with the physician, and after 2 years, 80% of schizophrenic patients discontinue neuroleptic treatment (Kemmler et al. 2005). The factors contributing to nonadherence include, i.e., poor illness insight, a negative attitude toward medication, substance abuse, and disorganization (Goff et al. 2011). In the long-term study CATIE, 74% of patients discontinued the treatment in the first year of the study (Lieberman et al. 2005). In EUFEST, 30% of patients stopped the treatment before the end of the study period (Kahn et al. 2008). The analysis of the combined results of the above studies shows that reduced adherence to pharmacological treatment was associated with substance use higher levels of hostility (Czobor et al. 2015).

The continuous treatment of schizophrenia is necessary to control the symptoms and prevent the relapse. The early discontinuation and irregular treatment result in relapses, drug intolerance, and drug resistance. Finding the ways to increase the drug compliance may change the situation and improve the prognosis in schizophrenia.

Different attempts are made to improve the compliance of schizophrenic patients. For example, an emphasis is put on psychoeducation (Bauml et al. 2016) or motivational interwieving (Fiszdon et al. 2016). Other factors that may lead to an improvement in this field are the therapeutic alliance, accessibility of care, and patients’ trust that the treatment will address their own unique goals (Dixon et al. 2016). The literature reports show that an important role in the succesful treatment of schizophrenia needs also a good collaboration with the primary care physicians (Jones et al. 2015), whose efforts can be supported by consulting psychiatrists working in the telepsychiatry paradigm (Johnston and Yellowlees 2016).

In the recent years, more and more evidence shows that telemedicine can be an effective method in treating psychiatric patients and improving their compliance (Hilty et al. 2013), and eMental health interventions have already become a standard in the treatment of psychotic disorders (Gaebel et al. 2016), which are highly accepted and tolerated by the patients (Mucic 2008). A growing body of evidence confirms that telepsychiatry is effective and produces reliable service outcomes similar to those provided in-person (i.e., face-to-face) (CADTH Rapid Response Reports 2015). The use of online tools reduces costs of the care provided to the patients. For this reason, telemedicine solutions will become an integral part of the whole health system, including psychiatric care. The possibility of clinical applications of telemedicine is very diverse. They are used when there are barriers to the flow of information between the patient and the healthcare professional and when access to information is essential to determine the proper procedure.

The aim of the study was to establish the treatment compliance level in schizophrenic patients and show how a telemedicine system may influence the compliance in schizophrenia. The group of schizophrenia patients in the stable symptomatic remission was chosen, expecting the compliance in this group to be good and assuming the group may benefit from the use of telepsychiatric platform.

## Subjects and Methods

The 12-month clinical study of the compliance in paranoid schizophrenia was performed on the group of 199 patients. The examined group consisted of paranoid schizophrenia patients, diagnosed according to ICD-10. All the patients recruited to the study were in the symptomatic remission (according to Andreasen et al. 2005). 57.3% of the group (*n* = 114) were males and 42.7% were females (*n* = 85); the mean age was 32 years (SD = 5.92). The patients were treated by the group of 45 psychiatrists, using tablets to contact patients on their smartphones. In order to exclude the potential confounding influence of drug effectiveness/tolerance, only patients with stable pharmacological treatment were recruited to the study. That means that in those patients, the treatment was not changed in the period of 1 month before the enrolment to the study and during that time, they are in a symptomatic remission. Every patient in the study was given a smartphone with the preinstalled original telemedicine platform (Telemedicine MoneoPlatform). The platform consists of three applications: tablet application for the physician, smartphone application for the patient, and an administrative application. The functionalities in the tablet application are the following: videoconferencing, compliance monitoring, cognitive training, video education, psychometric assessment, and electronic medical files recording. The smartphone application gives the patient the option to initiate the videoconference with the physician, watch the educational video recordings, perform cognitive trainings, and set the appointment in an outpatient clinic. All the functionalities, both in physician’s and patient’s applications, were used in the present study. The limitation of the study was, however, that not all confounding factors were controlled for.

Before the telemedicine platform was given to patients, it was validated for over 1 month in a group of ten selected psychiatrists with at least 5 years of clinical experience, who tested all the functionalities by playing both roles—of the patient and of the physician. The limitation of this process of validation could be the fact that it did not completely fulfill the criteria of an evaluation method. The testing scenarios were prepared to simulate different clinical situations. During the testing phase, the physicians assessed the reliability and functionality of all the platform functions. The tests were performed and evaluated repetitively once a week over the period of 6 weeks. The testing phase helped to validate the application as the reliable treatment tool before giving it to patients.

According to the therapeutic plan, introduced by the investigator on the visit in the outpatient clinic, the telemedicine platform kept sending to the patient reminders about the drug intake. Patients were reminded two times—first time 1 h before the planned drug intake and right on the time of the intake. The second communicate that appeared on patient’s smartphone was the question if they had taken the drug. The patient confirmed the drug intake by ticking the appropriate box on the touch screen. The telemedicine system recorded every confirmation and according to that, the compliance was counted as the percent of the doses confirmed in relation to the planned ones. At a monthly videoconference, the psychiatrist discussed the compliance record with the patients, and this feedback gave a possibility for a positive reinforcement for the study participants.

## Results

The treatment compliance in the study group was low (46%) from the very beginning of the study and has not improved over the study period.

The first significant finding of the study is the rate of compliance in the first month in the symptomatic remission patients. In the first month of the study, patients confirmed only 47.6% of the doses as taken. When analyzed in the total group, the compliance significantly decreased over the 12-month period (*p* < 0.001) (Table 1). After 12 months of the observation, the compliance decreased by 18.8%. The initial level of the treatment compliance had no impact on the obtained results. Both in the better compliance group (two higher quartiles) as well as in the worse compliance patients (two lower quartiles), the number of confirmed doses was decreasing significantly (*p* < 0.001).

**Table 1:** Descriptive statistics of the treatment compliance level in consecutive months of study

Month	Number	Mean (%)	SD (%)	Median (%)	Wilcoxon’s test^a^
1	136	47.6	39.1	45.7	–
2	142	45.4	36.1	44.0	*T* = 2377.0 *p* < 0.01
3	149	41.5	35.1	37.7	*T* = 2255.5 *p* < 0.001
4	149	39.4	35.0	32.5	*T* = 2176.5 *p* < 0.001
5	154	36.4	34.6	27.1	*T* = 2117.0 *p* < 0.0001
6	156	34.8	33.4	23.5	*T* = 1967.0 *p* < 0.0001
7	154	33.6	32.9	21.0	*T* = 1780.0 *p* < 0.0001
8	156	32.5	32.5	19.8	*T* = 1801.0 *p* < 0.0001
9	156	31.3	32.1	18.5	*T* = 1771.0 *p* < 0.0001
10	156	30.4	31.8	16.9	*T* = 1724.0 *p* < 0.0001
11	156	29.6	31.7	15.3	*T* = 1717.0 *p* < 0.0001
12	156	28.8	31.5	14.0	*T* = 1656.0 *p* < 0.0001

^a^Wilcoxon’s test compared the differences between the compliance after the first month to the compliance after the next months

## Discussion

To our knowledge, this project is the biggest long-term study of the treatment compliance in schizophrenia, performed on a large number of patients. The treatment compliance observed in our study group was low (46%) from the very beginning of the study and did not improve over the study period. This figure seems unsatisfactory on the one hand, as it is below 50%; on the other hand however, given the fact that it was a long-term study lasting 12 months with 199 active participants, our observations can be at least partly optimistic, as good compliance is often achieved in short-term studies with small number of participants and becomes much more difficult to achieve in long-term studies engaging larger study groups. The above observations are similar to those we made after analyzing the preliminary results following the first 6-month period; however, the main difference after 12 months was that we did not confirm our earlier finding that in the worse compliance of half of the patients, the adherence increased by 10.7% (Krzystanek et al. 2015). As mentioned above, after 12 months, both in the better compliance and in the worse compliance groups, the rate of adherence decreases significantly.

The success of telemedicine programs in psychiatry depends largely on the active participation of both patients and clinicians. So far, a discussion is being carried out, how to improve the adherence, especially in long-term studies, which often turns out not to be satisfactory. For example, Spaniel et al. (2015) report on their 18-month multi-center parallel randomized controlled, open label, trial ITAREPS targeted at 74 schizophrenic outpatients. According to the authors, this program failed to be successful in relapse prevention due to nonadherence of both psychiatrists and patients (Spaniel et al. 2015). The compliance of the patients looks much better in short-term programs with relatively small study groups. In the FOCUS intervention project by Ben-Zeev et al. (2014a, b), taking advantage of smartphones, the participants used the applications on 86.5% of days they had the device. The authors report that 38% of the use of the interventions was initiated by the participants, and 62% were a response to automated prompts (Ben-Zeev et al. 2014a). In another study focusing on dual diagnosis patients, in which the participants were expected to respond to text messages, a compliance ratio was relatively high, 87%; however, the study group was also quite small, 17, and the study lasted only 12 weeks (Ben-Zeev et al. 2014b). A good compliance followed by a significant reduction in emergency visits and medical appointments was also observed in the @HOME platform project by Frangou et al. (2005). The study group of 108 schizophrenic patients was significantly large; again, the duration of the study was not very long, only 8 weeks (Frangou et al. 2005). A positive observation relating to the adherence was done in a telemedicine study focusing on depressed patients, where it did not differ from the adherence in traditionally treated patients, although the group was relatively large, 119, and the study lasted 6 months (Ruskin et al. 2004). This difference in the observed results probably results from a difference in the type of psychiatric patients, whose general social functioning may be much better; anyway some optimistic conclusions can be drawn from them in terms of the usability of telemedicine approach.

In summary, the compliance of schizophrenic patients in telemedicine studies seems to be quite good in short-term studies involving relatively small groups of patients, but much more problems appear in long-term projects with larger study groups. The adherence depends then highly on the engagement of the patients and the investigators, and only this engagement can guarantee a success in such areas like clinical improvement, relapse prevention, and better social functioning. In this context, the compliance of 46% achieved in our study looks optimistic on the one hand but on the other hand raises a question: how we can improve it in our further projects?
